# A METHOD TO PREDICT AND UNDERSTAND FISH SURVIVAL UNDER DYNAMIC CHEMICAL STRESS USING STANDARD ECOTOXICITY DATA

**DOI:** 10.1002/etc.2144

**Published:** 2013-01-30

**Authors:** Roman Ashauer, Pernille Thorbek, Jacqui S Warinton, James R Wheeler, Steve Maund

**Affiliations:** † Environment Department, University of York, YorkUnited Kingdom; ‡ Eawag, Swiss Federal Institute of Aquatic Science and TechnologyDübendorf, Switzerland; § Syngenta, Environmental SafetyBerkshire, United Kingdom; ‖ Syngenta Crop Protection AGBasel, Switzerland

**Keywords:** Dose-response model, Time-variable exposure, Aquatic toxicity, Pesticide fate model, Carry-over toxicity

## Abstract

The authors present a method to predict fish survival under exposure to fluctuating concentrations and repeated pulses of a chemical stressor. The method is based on toxicokinetic-toxicodynamic modeling using the general unified threshold model of survival (GUTS) and calibrated using raw data from standard fish acute toxicity tests. The model was validated by predicting fry survival in a fish early life stage test. Application of the model was demonstrated by using Forum for Co-ordination of Pesticide Fate Models and Their Use surface water (FOCUS-SW) exposure patterns as model input and predicting the survival of fish over 485 d. Exposure patterns were also multiplied by factors of five and 10 to achieve higher exposure concentrations for fish survival predictions. Furthermore, the authors quantified how far the exposure profiles were below the onset of mortality by finding the corresponding exposure multiplication factor for each scenario. The authors calculated organism recovery times as additional characteristic of toxicity as well as number of peaks, interval length between peaks, and mean duration as additional characteristics of the exposure pattern. The authors also calculated which of the exposure patterns had the smallest and largest inherent potential toxicity. Sensitivity of the model to parameter changes depends on the exposure pattern and differs between GUTS individual tolerance and GUTS stochastic death. Possible uses of the additional information gained from modeling to inform risk assessment are discussed. Environ. Toxicol. Chem. 2013;32:954–965. © 2013 SETAC

## INTRODUCTION

Assessing the ecological risk of plant protection products (PPPs) requires the comparison of toxicity data for sensitive nontarget species with worst-case predicted exposure concentrations in the environment. Toxicity data used in these assessments usually are obtained from studies in which exposure concentrations are maintained for the duration of the experiments, usually lasting several days (for acute studies) to several weeks (for chronic studies). However, under realistic environmental conditions, concentrations of PPPs in aquatic systems will fluctuate, and exposure profiles may vary substantially 1–3. Risk assessments do not typically consider the nature of effects on nontarget species from fluctuating exposures that are likely to be encountered in the field; instead, peak or time-weighted average concentrations usually are used 4–6. To assess the environmental risk of PPPs, time series concentrations in surface water bodies are predicted using environmental fate models 3, 7. At present, it remains unclear how to appropriately integrate these exposure profiles with the effect assessments to refine the risk assessments 3.

Classical data analysis methods, more specifically concentration–effect curve models do not account for temporal aspects of exposure and toxicity 5, 8–11. However, in recent years alternative toxicity models that consider temporal aspects of toxicity have been developed 3, 9, 12. Toxicokinetic-toxicodynamic (TKTD) models simulate the time course of processes that lead to intoxication and can account for carry-over toxicity and delayed effects, which can be caused by slow elimination, slow organism recovery, or a combination of both processes 13. Moreover, they can process fluctuating or pulsed exposure concentrations as an input to predict survival over time. Thus, they are well-suited for risk assessment of dynamic chemical stress 3, 9. Recently, TKTD models for survival have been unified and integrated in the general unified threshold model of survival (GUTS) 8.

Standard toxicity tests are typically carried out with maintained exposures, whereas measured and simulated exposure to PPPs in water bodies generally occur in fluctuating and highly variable patterns 1–4, 7. Thus, toxicity must be extrapolated from relatively constant to relatively variable exposure profiles. Toxicokinetic-toxicodynamic models simulate the time course of processes leading to toxicity, including processes that cause carry-over toxicity or delayed effects 9, 13. Carry-over toxicity can be caused by bioaccumulation and slow elimination, as well as slow toxicodynamic (TD) organism recovery 8, 13. The ability to model carry-over toxicity is the key reason why greater confidence can be placed in toxicity extrapolations for fluctuating or pulsed exposure patterns based on TKTD models rather than on predictions based on time-weighted averages 8, 9, 13, 14. Furthermore, TKTD model parameters have a mechanistic interpretation and can thus be evaluated with other studies, such as bioaccumulation tests. Toxicokinetic (TK)models have been demonstrated to accurately predict internal concentrations in fish 15. Several reviews of the extrapolation of toxicity to fluctuating exposures are available 5, 9, 16, 17, and some studies have investigated the calibration and predictive power of TKTD models for survival 14, 18, 19. Thus, TKTD models offer options for higher-tier risk assessment tools that can be used for robust evaluations of likely effects resulting from time-varying exposure, which is likely to be the rule rather than the exception.

Here, we present a case study for a PPP in which fish survival under fluctuating concentrations is simulated using a TKTD model. The model is first calibrated using standard laboratory data from acute fish toxicity studies and is then validated by comparing the predicted survival with that from an independent experiment. Predicted surface water concentration profiles for the PPP are generated using the Forum for Co-ordination of Pesticide Fate Models and Their Use surface water (FOCUS-SW) model 7. Application of the TKTD model is demonstrated by using these FOCUS-SW exposure patterns as model input and predicting fish survival for a 485-d period from the exposure modeling. The objective of the present study is to demonstrate the method, illustrate what kind of information can be derived, and indicate how that additional evidence can be used to inform and refine environmental risk assessments.

## MATERIALS AND METHODS

### Test substance

The present study was carried out with benzovindiflupyr, a broad-spectrum pyrazole carboxamide fungicide (fungicidal mode of action via succinate dehydrogenase inhibition [SDHI], causing respiration inhibition at complex II).

### Toxicity data

Fish acute toxicity data were generated using the Organisation for Economic Co-operation and Development (OECD) test guideline 203 with carp (*Cyprinus carpio*) and fathead minnow (*Pimephales promelas*). These two species were the most sensitive of five tested (see *Discussion* and Supplemental Data), with 96-hour median lethal concentrations (LC50) of 3.5 and 4.7 µg/L for carp and fathead minnow, respectively. Raw survival data for daily survival, mean measured exposure concentrations, and control mortality data were used for the modeling.

### Exposure data

Predicted surface water concentration profiles were generated using the FOCUS-SW models 7. Simulations were for one and two applications to cereals (spring and winter) at 75 g active substance per hectare with a 14-d interval between applications. Six exposure profiles were selected from all the relevant cereal scenarios for analysis with TKTD models. These were the FOCUS-SW drainage scenarios D1 ditch, D1 stream, D2 ditch, D2 stream, D4 stream, and D6 ditch (see 7 for detailed descriptions). These profiles cover the worst-case, or extreme worst-case temperature, soil, and hydrology conditions for surface water exposure via drainage and consequently the highest predicted concentrations for the European Union scenarios. The complete 485-d water concentration–time series generated from the FOCUS-SW model TOXic substances in Surface WAters (TOXSWA) was used as the time-variable exposure input for the TKTD modeling of fish survival.

### GUTS

A framework for TKTD modeling has been recently published that unifies all previously used TKTD models for survival 8. This GUTS can be reduced to two special cases, which are based on the assumption of either individual tolerance distribution (GUTS-IT) or stochastic death (GUTS-SD) 8, 19. These two extreme cases of the GUTS were both used with the scaled internal concentration (see *Scaled internal concentration* section, below) as the dose metric 8. The two cases can be derived for the assumption of SD and the assumption of IT, whereas the “real” behavior is likely somewhere in between. In other words, these two special cases provide the boundaries of the “real” time course of toxicity that would be predicted by a more complex and difficult-to-calibrate mixed SD and IT model (see Jager et al. 8).

The two models GUTS-SD and GUTS-IT are described briefly below (see *GUTS-SD* and *GUTS-IT* sections). For a full explanation please see Jager et al. 8. A non-mathematical review is described in Ashauer and Escher 9. Note, however, that the GUTS-SD and GUTS-IT models do not require any a priori assumptions about the speed of compound elimination or the speed of organism recovery. These models let the data speak and capture information on the time course of toxicity in their model parameters during the calibration step. This is an important improvement over previous TKTD models, such as critical body residues or critical target occupation, which required a priori assumptions on the speed of recovery. These previous models are unified in GUTS, which is currently the most general and rigorous framework to predict survival over time.

### Scaled internal concentration

The scaled internal concentration is defined as the internal concentration divided by the bioconcentration factor (the ratio of uptake rate constant and elimination rate constant). Its plot over time follows the same time course as the internal concentration; however, the calculation of the scaled internal concentration requires only one parameter. In cases where the time course of internal concentrations is not measured, then the scaled internal concentration should be used 8


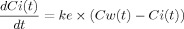
(1)

where *Ci*(*t*) is the time course of the scaled internal concentration (µg/L), *t* is time (day), *Cw*(*t*) is the time course of the concentration in water (µg/L) and *ke* is the dominant rate constant (1/d). The dominant rate constant quantifies the elimination of the substance (TK) and the organism recovery (TD). We fit the parameters of Equation 1 using the time course of fish survival data (see *Model calibration* section below) because measured internal concentrations are not available. In this case, the time course of the scaled internal concentration captures both the time course of toxicant elimination and the time course of organism recovery. Both processes (TK and TD) potentially play a role, but we cannot differentiate between them without measured internal concentrations and approximate the two-compartment system by a one-compartment system. The time course of toxic effects will be dominated by the slower of these two processes. Thus, rather than elimination rate constant or organism recovery rate constant, the parameter *ke* is termed the dominant rate constant and reflects the time course of TK and TD combined 8, 19. For the same reasons, *Ci(t)*, which we use as the dose metric, reflects the time course of internal concentrations of the substance and the time course of damage it causes (see Jager et al. 8 for a detailed discussion).

### GUTS-SD

The instantaneous probability for an organism to die is the hazard rate 8. For GUTS-SD, the hazard rate is calculated as


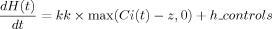
(2)

where d*H*(t)/dt is the hazard rate (1/d), *kk* is the killing rate constant (L/[µg × d]), *Ci*(t) is the time course of the scaled internal concentration (µg/L), *t* is time (day), z is the threshold (µg/L) and *h_controls* is the background hazard rate (control mortality rate, 1/d). The GUTS-SD assumes that all individuals in the test population have the same threshold, *z* (see also Jager et al. 8 and Nyman et al. 19). The survival probability—that is, the probability of an individual to survive until time *t—*is given by


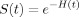
(3)

where *S(t)* is the survival probability (−).

### GUTS-IT

The GUTS-IT model assumes that the value of the threshold *z* differs among individuals in the test population and follows a distribution (see also Jager et al. 8 and Nyman et al. 19). When the threshold is exceeded, it is assumed that the individual dies immediately. Here, GUTS-IT also uses the scaled internal concentration as dose metric (Eq. 1) and the threshold *z* is the scaled internal concentration above which the individual dies instantly. Thus, the number of deaths over time can be calculated based on the distribution of the threshold *z* in the population. The cumulative log-logistic distribution of the threshold *z* is calculated as


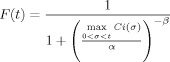
(4)

where *F(t)* is the cumulative log-logistic distribution of the threshold *z* over time (−), *Ci*(*σ*) is the time course of the scaled internal concentration (µg/L), *t* is time (day), *σ* (day) is time before the current point in time *t*, *α* is the median of the distribution of *z* (µg/L), and *β* is the shape parameter of the distribution of *z* (−). The survival probability—that is, the probability that an individual will survive until time *t*—is given by


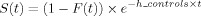
(5)

where *S(t)* is the survival probability (−) and *h_controls* is the background hazard rate (control mortality rate, 1/d).

### GUTS-SD and GUTS-IT in multiple pulse exposures

Due to their inherent assumptions, the models GUTS-SD and GUTS-IT result in different survival predictions for repeated pulse exposures. The SD approach assumes that death is a chance process; thus, the proportion of a population that is killed by a series of identical pulses remains constant, irrespective of previous exposure. The IT approach assumes that there is a distribution of tolerances in the exposed population so that tolerant individuals will not be affected by repeated exposure to the same (or lower) toxicant concentrations. To illustrate these assumptions, consider a hypothetical example with two identical pulses in sequence, where the first pulse kills 50 out of 100 fish. Given that the interval between the two pulses is long enough for complete elimination of the substance and organism recovery, GUTS-SD would predict that the second pulse kills 25 fish, whereas GUTS-IT would predict that the second pulse kills zero fish. Mortality is a chance process in GUTS-SD, with all fish in the population having equal probabilities to die and the same probability applies at the first and second pulse. Mortality is a deterministic process in GUTS-IT, following a distribution of thresholds in the fish population, where the first pulse kills the more sensitive fraction of all fish and the strong individuals survive both the first and the second pulses. Thus, it is important to use both models together (for detailed discussions see 8, 20, 21).

### Model calibration

Raw data from the carp and fathead minnow toxicity tests were used in the model calibration. Mean measured concentrations served as model input, whereas survival data for each day and control mortality data served as calibration data. In addition to the toxicity studies, a fish bioconcentration (OECD test guideline 305) test with bluegill sunfish (*Lepomis macrochirus*) was available. The depuration rate constant (*kd* = 1.2802 1/d) from the bioconcentration study was used as initial value for *ke* in the model calibration for carp, which was the first species for which we calibrated the TKTD model.

The parameters for GUTS-SD and GUTS-IT were found by maximizing the log likelihood function (see Jager et al. 8 for derivation)


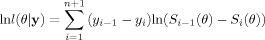
(6)

where *l* is the likelihood, *y* is the time series of the number of survivors, *i* is sampling date, *n* is the number of sampling dates, θ is the vector of model parameters and *S*(θ) is the survival probability given θ. The likelihood was calculated for each treatment (test concentration in the acute toxicity test) and the ln likelihoods for all treatments were summed. We then used the downhill simplex algorithm to find the minimum -ln likelihood in parameter space. The parameter values at the smallest -ln likelihood are the best fit parameter values and are used to predict survival in other exposure scenarios. Thus, information about the time course of toxicity is extracted from the acute toxicity test data, captured by the model and its parameter values, and then used to predict survival with other concentration time series as input (e.g., FOCUS-SW scenarios).

The parameters *ke*, *kk*, and *z* were calibrated for the GUTS-SD model, and the parameters *ke*, *α*, and β were calibrated for the GUTS-IT model. The confidence limits were calculated by profiling the likelihood 22 for each parameter separately. The 95% confidence limits are reported. The mortality in the control groups was used to calibrate the background hazard rate (i.e., approximate background mortality). Both the control and solvent control survival data were combined for that analysis. The resulting control mortality rate *h_controls* was 0.018 (1/d) with confidence limits (0.001, 0.032) for carp. The survival data for control fish in the acute toxicity test with fathead minnow did not show any mortality. Because a background mortality rate of zero is biologically impossible and causes computational problems during calibration, the control mortality rate *h_controls* was set to 0.00315 (1/d) for this species. This control mortality rate corresponds to 10% survival over two years (730 d), reflecting the natural life span of the fathead minnow. The control mortality rate was kept fixed when calibrating the other model parameters.

### Model testing (validation)

A fish early life stage study (ELS; OECD test guideline 210 23) with fathead minnow was available to independently test the model predictions with data other than the acute toxicity test results. Survival of fry in the ELS study was compared to survival predicted with GUTS-SD and GUTS-IT. In the ELS study, testing was initiated with freshly fertilized embryos, which were exposed for approximately 4 d until fry hatch and then until 28 d posthatch. Survival was recorded daily over the 32-d exposure period. The survival of fry during the 28 d after hatching was predicted with GUTS-SD and GUTS-IT using the exposure concentrations in the ELS study as input. Nominal concentrations were used, because measured concentrations were sufficiently close to nominal. The exposure of embryos was not taken into account in the modeling, because the change from embryos to fry cannot be modeled with GUTS. Model simulations therefore started after hatching, not taking into account any potential toxicity resulting from pre-exposure of embryos. The background mortality in the ELS study was simulated based on fry survival in the controls. Because the model was calibrated with data from the older life stages used in standard acute toxicity tests but tested against data for fry survival (generally considered a more sensitive life-stage), this evaluation would provide insights into how conservative the model predictions are likely to be.

### Model predictions for realistic field exposure patterns

First, fish survival was predicted using the exposure time series (hourly time step) from FOCUS-SW simulations directly as input. The control mortality rate *h_controls* was set to zero for the predictions of survival, because the interest was solely the effect of the active substance on fish. The concentration time series (total mass concentration of the substance in the water column) of the TOXSWA simulations (file: *.cwa) was converted to µg/L and used as input for the simulations with GUTS-SD and GUTS-IT. Fish survival in these concentration patterns was then simulated using the best fit parameters for GUTS-SD and GUTS-IT, respectively.

Second, we simulated fish survival in FOCUS-SW exposure patterns where the exposure concentrations were multiplied by a standard factor to generate higher exposure concentrations. The concentrations were multiplied with an exposure multiplication factor of five or 10 and used as input for the simulations with GUTS-SD and GUTS-IT. The purpose of this factor was to gain some insight into the magnitude of difference between the predicted exposure concentrations and those that would be likely to cause mortality. For example, if an exposure scenario five or 10 times greater than the worst-case FOCUS exposures resulted in limited mortality predictions, it would provide further reassurance that mortality from field exposure was unlikely to occur.

### Margin of safety calculation

When the concentration profiles from TOXSWA are used directly as input for the survival simulation, survival is 100% in all surface water scenario exposure simulations. If the simulated survival in the FOCUS-SW exposure patterns is 100%, even when the concentrations are multiplied by a factor of five or 10, it remains unclear how far these fluctuating concentrations are below concentrations that might cause the onset of mortality. To understand the potential hazard from the fluctuating exposure profiles, it is desirable to quantify how far these concentrations are below levels that cause mortality. This margin of safety can be quantified separately for each exposure pattern by finding the exposure multiplication factor that corresponds to the onset of mortality.

A practical definition of the onset of mortality is thus required. In the present study, as a pragmatic solution, we chose 10% mortality over 485 d as a proxy for the onset of mortality. Thus, each FOCUS-SW concentration profile was multiplied by a factor such that the survival simulation would result in 10% mortality at the end of the simulation period (485 d). These factors were calculated separately for each scenario and each model (GUTS-SD and GUTS-IT). Essentially, a data point was defined for 90% survival at day 485 and then the model was calibrated to fit that data point by changing the parameter factor. This factor characterizes the margin of safety for each scenario; that is, it quantifies how far the exposure profiles are below toxic concentrations. The calculation of the factor using the TKTD model takes the fluctuating nature of the concentration profiles into account and accounts for carry-over toxicity.

### Organism recovery times

Organism recovery times quantify the time that an organism needs to recover from previous exposure so that carry-over toxicity does not occur 13, 24. This time is characteristic for each combination of species and toxicant and can be calculated as the time that the dose metric (in the present study, *Ci(t)*) needs until it has declined below 5% of its maximum after a pulsed exposure. Previous studies have used a 1-d pulsed duration followed by a sufficiently long postexposure period. The concentration of the 1-d pulse exposure was chosen such that the pulse eventually kills (including the postexposure period) 50% of the test population 13, 19, 24. We simulated such a pulse and monitored the increase of the scaled internal concentration during the exposure and its subsequent decrease. This simulation yields the time (organism recovery time) in which the scaled internal concentration declines below 5% of its maximum after a 1-d pulse that eventually kills 50%. Because the scaled internal concentration in our one-compartment model represents the TK and TD, the organism recovery time measures how fast the combined depuration of the substance and recovery of damage is. The organism recovery time indicates how long the interval between subsequent pulses should be before they can be viewed as toxicologically independent exposure events. The organism recovery time only depends on the dominant rate constant *ke* and reflects combined TK and TD recovery.

### Comparing the toxicity potential of different exposure patterns

We calculated the areas under the exposure curves using the concentrations of each FOCUS-SW scenario multiplied with its respective margin of safety (as previously defined). This ensures that all exposure profiles in the comparison result in the same effect (10% mortality after 485 d), and the differences in the areas under the exposure curves characterize which patterns are more or less toxic. Because all patterns result in the same effect, patterns with lower areas under the exposure curve are inherently more toxic, and patterns with larger areas under the exposure curves are inherently less toxic. Thus, the toxicity potential of the different exposure patterns can be compared by calculating the area under the exposure concentration curve. We also calculated three additional characteristics of each exposure pattern that help to understand the toxicity potential: number of peaks, peak duration, and distance between peaks. This calculation uses a previously published stepwise classification algorithm to identify peaks in the exposure time series 25.

### Sensitivity and uncertainty analysis

A sensitivity and uncertainty analysis was carried out by varying each model parameter separately within its confidence limits. A Monte Carlo simulation (1,000 runs) was set up with uniform distributions ranging from the lower to the upper confidence limit for each parameter. These simulations were carried out with GUTS-SD and GUTS-IT for fathead minnow and the FOCUS-SW exposure profiles with the highest (D4 stream) and the lowest (D2 ditch) inherent toxic potential. Each scenario was simulated with the exposure multiplication factor set to the respective margin of safety such that the simulation with the best fit parameter values would result in 90% survival over 485 d. The survival after 485 d was recorded for each Monte Carlo run and plotted against the percentage variation in the parameter value.

### Model implementation

The models were implemented and run in ModelMaker (Version 4; Cherwell Scientific). The code of the model implementation has been thoroughly checked and tested and has been used extensively in scientific research (see, for example, Nyman et al. 19).

## RESULTS

### Calibration

**Fig. 1:**
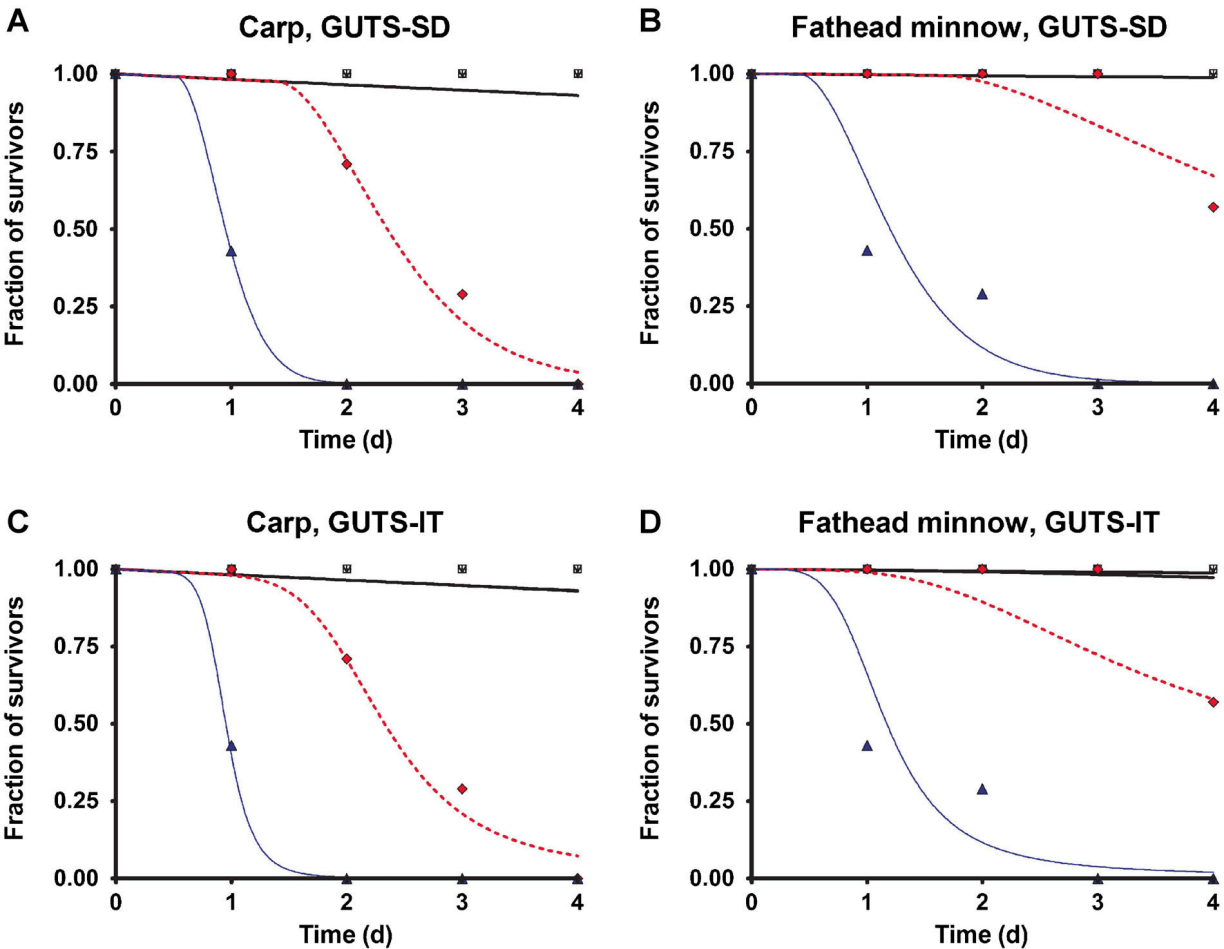
Model fit of general unified threshold model of survival–stochastic death (GUTS-SD; **A**,**B**) and general unified threshold model of survival–individual tolerance (GUTS-IT; **C**,**D**) 8 to the raw data from acute toxicity tests with carp (**A**,**C**) and fathead minnow (**B**,**D**). In both experiments, only the two highest concentrations yielded mortalities. These concentrations were 5.4 µg/L (dotted line, diamonds) and 10 µg/L (solid line, triangles) for carp and 4.4 µg/L (dotted line, diamonds) and 9.4 µg/L (solid line, triangles) for fathead minnow. [Color figure can be seen in the online version of this article, available at http://wileyonlinelibrary.com]

The parameter values of the best fit are given in Table 1 together with the corresponding likelihood value. The fitted models are plotted together with the raw data from the acute toxicity tests in Figure 1. Parameters converged to plausible best fit parameter values for both fish species and both the GUTS-SD and the GUTS-IT models without the need for parameter constraints. These parameter values were used for the predictive simulations with the concentration profiles from FOCUS-SW scenarios. Profiles of the likelihood were calculated for each parameter to derive the confidence limits. These likelihood profiles served as further checks on the convergence of the model fit.

**Table 1:** Parameter values of the two limit cases of the general unified threshold model for survival (GUTS), stochastic death (SD) and individual tolerance (IT), after calibration to raw data from the fish acute toxicity test

Parameter	Units	Best fit value	95% confidence limits
GUTS-SD, carp (–∑ln likelihood = 14.5694, *h_controls* = 0.018 [1/d])			
*ke*	1/d	1.00	0.82, 1.29
*kk*	L/(µg × d)	1.42	0.72, 2.52
*z*	µg/L	4.06	3.58, 4.42
GUTS-IT, carp (–∑ln likelihood = 15.0071, *h_controls* = 0.018 [1/d])			
*ke*	1/d	0.47	0.40, 0.54
α	µg/L	3.63	3.30, 4.03
β	–	10.52	5.60, 21.9
GUTS-SD, fathead minnow (–∑ln likelihood = 16.7797, *h_controls* = 0.0035 [1/d])			
*ke*	1/d	1.28	0.87, 2.64
*kk*	L/(µg × d)	0.42	0.20, 0.77
*z*	µg/L	3.85	3.25, 4.20
GUTS-IT, fathead minnow (–∑ln likelihood = 18.5279, *h_controls* = 0.0035 [1/d])			
*ke*	1/d	0.47	0.34, 0.65
α	µg/L	3.97	3.33, 4.81
β	–	5.54	2.85, 9.54

In addition to fitting GUTS-SD and GUTS-IT to data from carp and fathead minnow as reported in the present study, we also fitted the two models to acute toxicity data from three additional fish species (*Oncorynchus mykiss, Cyprinodon variegatus,* and *Lepomis macrochirus*), which were less sensitive than carp and fathead minnow. The model parameters for these additional fish species can be found in the Supplemental Data.

### Model testing (validation)

**Fig. 2:**
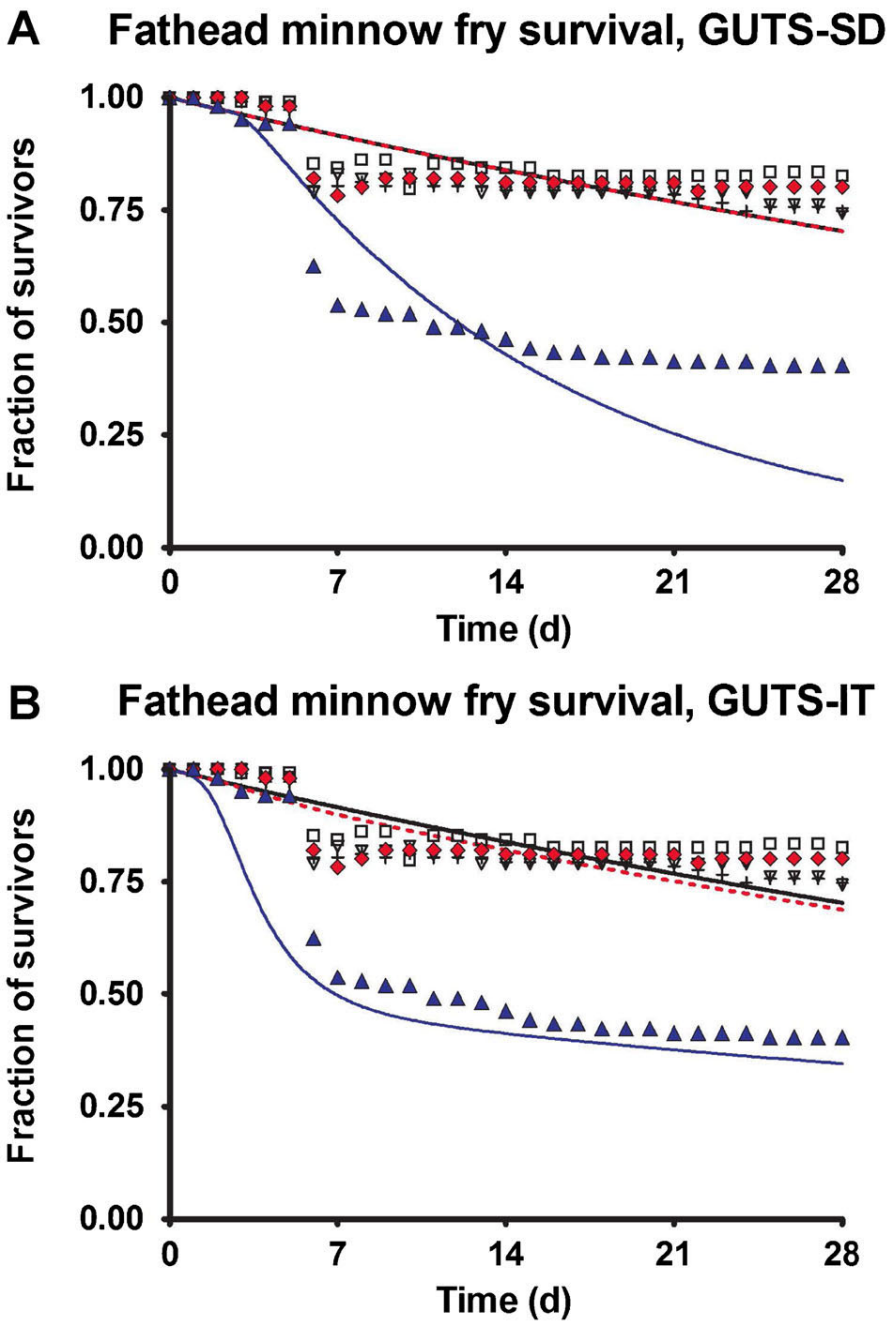
Prediction of fry survival in the fathead minnow early life stage study. The model predicts mortality in excess of controls for the treatment with 4 µg/L (solid line, triangles), the highest tested concentration. There is a typical drop in survival between days 5 and 6, across treatment groups, as larvae transition to free feeding. GUTS-SD and GUTS-IT = the two limit cases of the general unified threshold model for survival (GUTS), including stochastic death (SD) and individual tolerance (IT) 8. [Color figure can be seen in the online version of this article, available at http://wileyonlinelibrary.com]

The predicted survival of fathead minnow fry in the ELS study is shown in Figure 2. Survival in the treatments with the four lower concentrations is similar to the control mortality. GUTS-IT predicted only background mortality for these treatments, whereas GUTS-SD predicts a very small fraction of additional mortality in the second-highest concentration (see Figure 2, diamonds for data and dotted line for model prediction). The treatment with the highest concentration (4 µg/L) showed increased mortality, which was predicted with very good visual agreement with the GUTS-IT model. The GUTS-SD model also predicted mortality in the highest treatment, although with a different time course and pattern than observed.

### Predicted survival in FOCUS-SW scenarios

Six FOCUS-SW scenarios were analyzed in combination with two models (GUTS-IT and GUTS-SD), two species (carp and fathead minnow) and the two application patterns (one or two applications), resulting in 48 combinations. All 48 simulations using the TOXSWA output directly (realistic simulation) resulted in 100% survival for all scenarios, fish species, and both models. Thus, it can be concluded that no mortalities are expected if fish are exposed to these concentration profiles. All 48 simulations using the TOXSWA output multiplied with an exposure multiplication factor of five resulted in 100% survival for all scenarios and fish species with both GUTS-IT and GUTS-SD. Thus, if an exposure multiplication factor of five was applied, there would be no mortality in any of the combinations of fish species, FOCUS-SW scenarios and models.

**Fig. 3:**
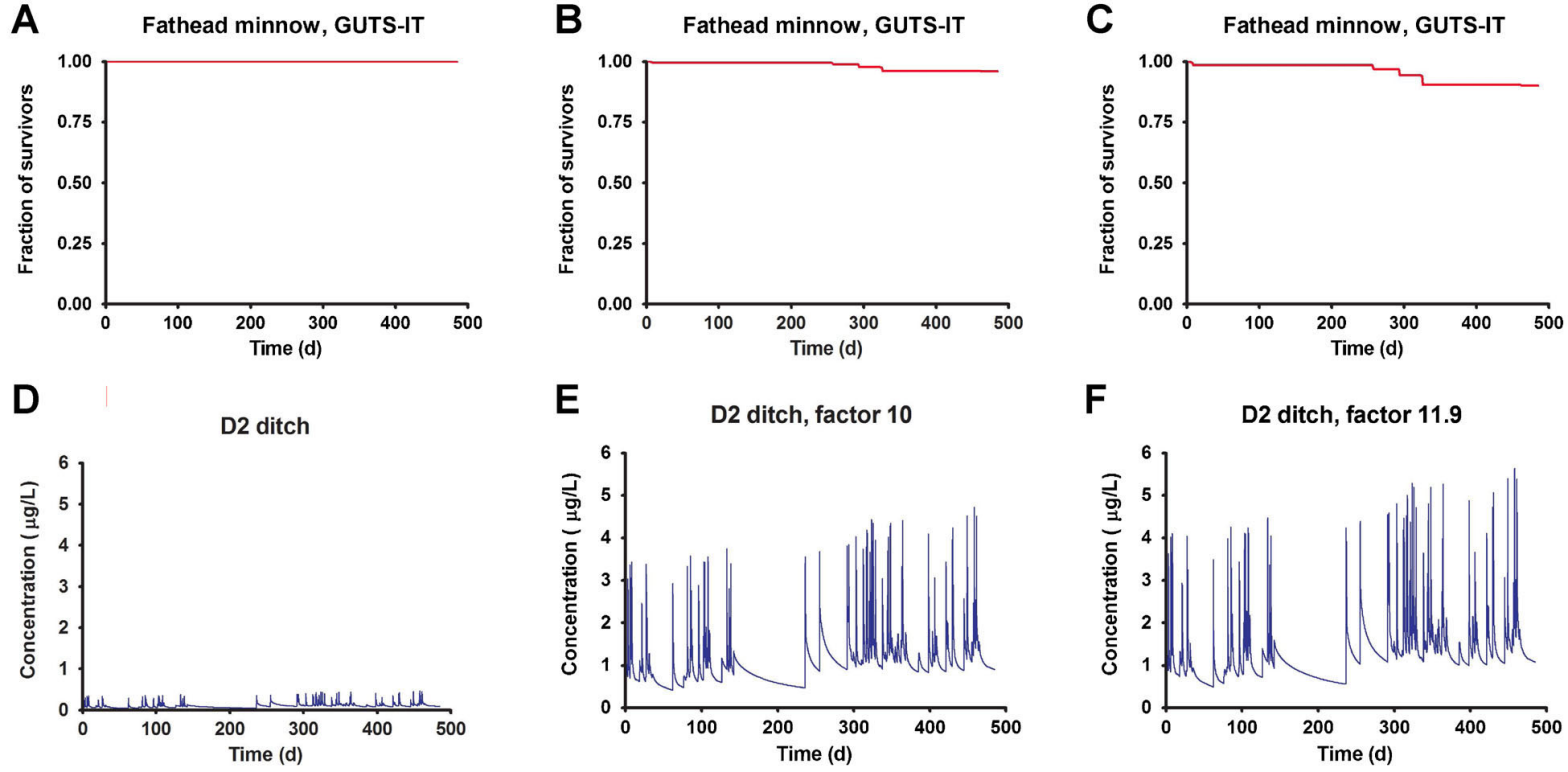
Illustration of method: Simulations of fathead minnow survival with GUTS-IT model in FOCUS-SW model D2 ditch (**A**,**D**), in D2 ditch exposure concentrations multiplied with factor 10 (**B**,**E**), and in D2 ditch exposure concentrations multiplied with factor 11.9 (margin of safety, 90% survival over 485 d; **C**,**F**). Graphs show predicted survival (**A-C**) and concentrations in the water body (**D**–**F**). FOCUS-SW = Forum for Coordination of Pesticide Fate Models and Their Use (pesticide fate models used for environmental risk assessment in Europe) 7. See Figure 2 caption for definition of GUTS-IT. [Color figure can be seen in the online version of this article, available at http://wileyonlinelibrary.com]

The simulations using the TOXSWA output multiplied with an exposure multiplication factor of 10 resulted in three of the 48 combinations with less than 100% survival (Table 2). These three cases, all with the GUTS-IT model and two PPP applications, were 99% survival of carp in D2 ditch and 99% and 96% survival of fathead minnow in D1 ditch and D2 ditch, respectively. This indicates that even at exposures an order of magnitude greater than the worst-case FOCUS exposure concentrations, the levels of mortality expected would be no level of mortality to very low levels. The lack of mortality indicated in the evaluation of the FOCUS scenarios themselves is likely to be a robust conclusion. Figure 3 shows the example of fathead minnow survival simulated with GUTS-IT, where the two middle panels (Fig. 3B and E) illustrate the analysis using a factor of 10.

**Table 2:** Results of simulated fish survival in FOCUS-SW exposure patterns^a^

Scenario^b^	Model (GUTS)	Carp two applications	Carp two applications	Carp one application	Carp one application	Fathead minnow two applications	Fathead minnow two applications	Fathead minnow one application	Fathead minnow one application

Endpoint		Survival in 10× higher concentrations (at day 485)	Factor (safety margin)	Survival in 10× higher concentrations (at day 485)	Factor (safety margin)	Survival in 10× higher concentrations (at day 485)	Factor (safety margin)	Survival in 10× higher concentrations (at day 485)	Factor (safety margin)
D1 ditch	SD	100%	21.4	100%	52.4	100%	20.5	100%	49.5
	IT	100%	16.3	100%	41.1	99%	14.8	100%	37.3
D1 stream	SD	100%	34.2	100%	85.9	100%	32.9	100%	82.3
	IT	100%	26.1	100%	65.9	100%	23.6	100%	59.7
D2 ditch	SD	100%	16.3	100%	36.4	100%	15.3	100%	34.0
	IT	99%	13.2	100%	29.5	96%	11.9	100%	26.8
D2 stream	SD	100%	28.3	100%	53.6	100%	26.4	100%	50.7
	IT	100%	23.2	100%	41.3	100%	21.0	100%	37.5
D4 stream	SD	100%	80.0	100%	177	100%	74.7	100%	165
	IT	100%	82.7	100%	184	100%	74.9	100%	167
D6 ditch	SD	100%	28.6	100%	61.8	100%	25.5	100%	55.1
	IT	100%	30.6	100%	66.8	100%	27.8	100%	60.5

a FOCUS-SW are the Forum for Co-ordination of Pesticide Fate Models and Their Use (FOCUS) surface water models [7] (pesticide fate models used for environmental risk assessment of pesticides in Europe).

b Each scenario consists of a set of environmental boundary conditions (e.g., soil, weather) for pesticide fate simulations that reflect realistic worst-case landscapes in Europe.

GUTS = general unified threshold model for survival; SD = stochastic death; IT = individual tolerance.

### Margin of safety

**Fig. 4:**
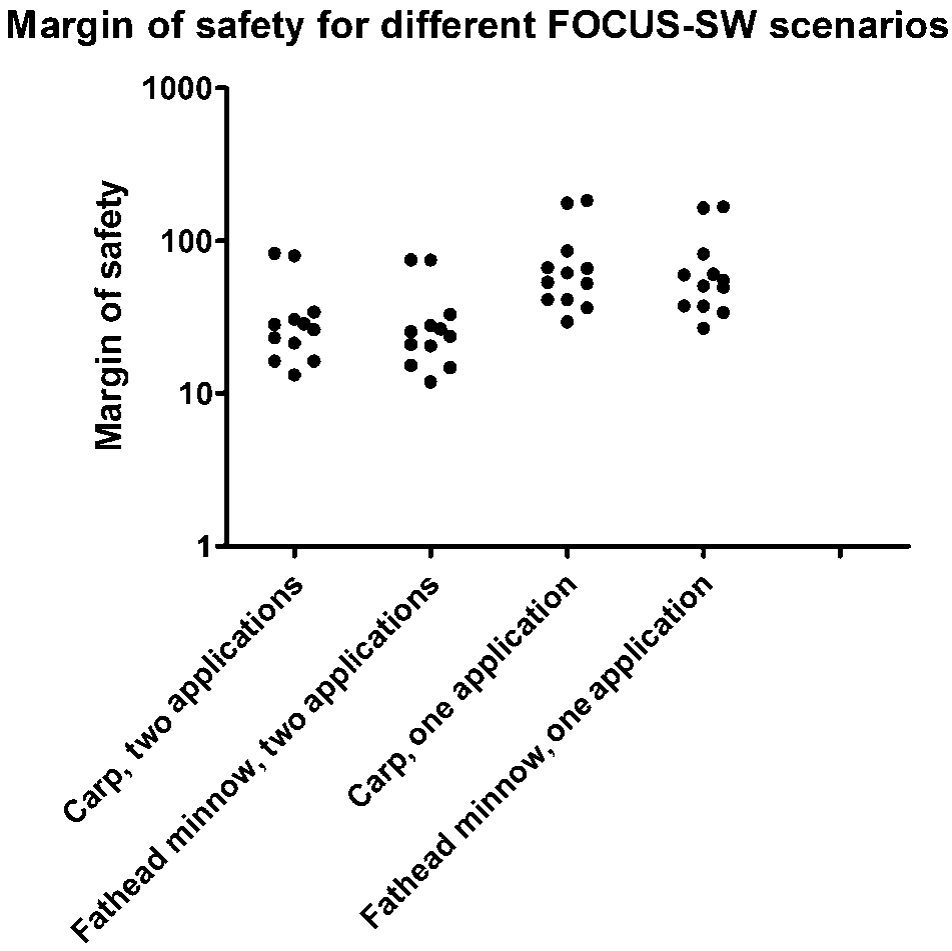
Margins of safety quantify how far below toxic levels the exposure patterns are. Each group consists of 12 data points that result from simulations for the six FOCUS-SW scenarios analyzed with two models (GUTS-SD and GUTS-IT). See Figures 2 and 3 caption for definitions of FOCUS-SW, GUTS-SD, and GUTS-IT.

The safety margins ranged from a factor of 12 to 184 (Table 2). This demonstrates that the concentrations in the original FOCUS-SW exposure profiles are a factor 12 to 184 below the levels where mortality might be expected in the analyzed combinations of fish species and application patterns. The distribution of the safety margins is plotted in Figure 4, illustrating their range. Figure 3C and 3F illustrates survival of fathead minnow simulated with GUTS-IT and a factor of 11.9, which is the margin of safety for this combination of species, scenario, and model.

### Organism recovery times

The organism recovery times were calculated after a 1-d pulse. The organism recovery times were 6.4 d for both carp and fathead minnow in the GUTS-IT model. They are identical because both species have the same value of the dominant rate constant in GUTS-IT. For GUTS-SD, the organism recovery times were 3.0 d for carp and 2.4 d for fathead minnow.

### Comparing toxicity potential of different exposure patterns

**Fig. 5:**
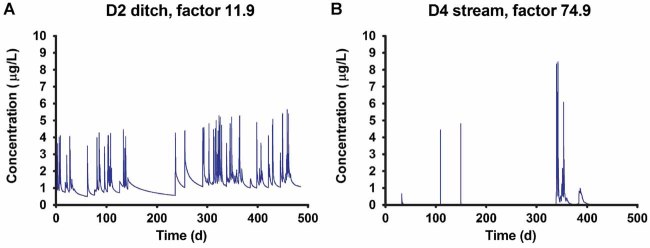
Comparison of the exposure pattern with the smallest inherent toxic potential (D2 ditch; **A**) with the exposure pattern that has the highest inherent toxic potential (D4 stream; **B**). Both patterns were multiplied with their respective margins of safety (factor) so that they result in the same effect (10% mortality after 485 d for fathead minnow in GUTS-IT). Both patterns result from two applications. See Figure 2 caption for definition of GUTS-IT. [Color figure can be seen in the online version of this article, available at http://wileyonlinelibrary.com]

The areas under the exposure curves for the different FOCUS-SW scenarios are shown in Table 3. All exposure patterns were multiplied with their respective margin of safety to result in 10% mortality after 485 d. The scenario D2 ditch had the lowest inherent toxic potential because it requires the largest area under the exposure curves to achieve 10% mortality. In contrast, D4 stream had the highest inherent toxic potential because it required the lowest area under the exposure curve to achieve 10% mortality. In other words, if the PPP was applied in application rates such that scenarios D2 ditch and D4 stream had the same area under the exposure curves, but preserved the current shapes of the concentration time series, then D2 ditch would be the least toxic and D4 stream would be the most toxic concentration time series. Both FOCUS-SW scenarios are shown in Figure 5, and Table 4 summarizes the additional characteristics calculated for each exposure pattern.

**Table 3:** Area under the exposure curve (∫Cwater) for the different exposure profiles (in µg × d/L) when they are multiplied with their respective safety margins^a^

Scenario^b^		Carp two applications	Carp one application	Fathead minnow two applications	Fathead minnow one application
					
Exposure	Model (GUTS) c	µg × d/L	µg × d/L	µg × d/L	µg × d/L
D1 ditch	SD	822.5	770.8	789.0	727.5
	IT	625.8	604.9	567.3	548.3
D1 stream	SD	650.7	621.8	624.4	596.0
	IT	495.7	477.3	449.3	432.6
D2 ditch	SD	847.7	801.3	792.3	749.9
	IT	683.6	650.0	619.7	589.2
D2 stream	SD	724.7	606.0	676.7	572.5
	IT	593.0	466.8	537.5	423.1
D4 stream	SD	42.3	38.9	39.5	36.3
	IT	43.7	40.5	39.6	36.8
D6 ditch	SD	86.1	76.3	76.8	68.1
	IT	92.2	82.5	83.5	74.7

a All exposures compared here result in 90% survival after 485 d.

b General unified threshold model for survival (GUTS), stochastic death (SD) and individual tolerance (IT), are the two limit cases 8.

c Each scenario consists of a set of environmental boundary conditions (e.g., soil, weather) for pesticide fate simulations that reflect realistic worst-case landscapes in Europe. The scenarios are part of the Forum for Co-ordination of Pesticide Fate Models and Their Use surface water (FOCUS-SW) models 7, pesticide fate models used for environmental risk assessment of pesticides in Europe.

**Table 4:** Additional characteristics of each exposure profile

Scenario^a^	Number of peaks	Mean interval between peaks, days	Mean duration of peaks, days
D1 ditch	20	22	19
D1 stream	30	15	12
D2 ditch	73	6	6
D2 stream	109	4	3
D4 stream	17	22	8
D6 ditch	20	22	19

a Each scenario consists of a set of environmental boundary conditions (e.g., soil, weather) for pesticide fate simulations that reflect realistic worst-case landscapes in Europe. The scenarios are part of the Forum for Co-ordination of Pesticide Fate Models and Their Use surface water (FOCUS-SW) models 7, pesticide fate models used for environmental risk assessment of pesticides in Europe.

### Sensitivity and uncertainty analysis

**Fig. 6:**
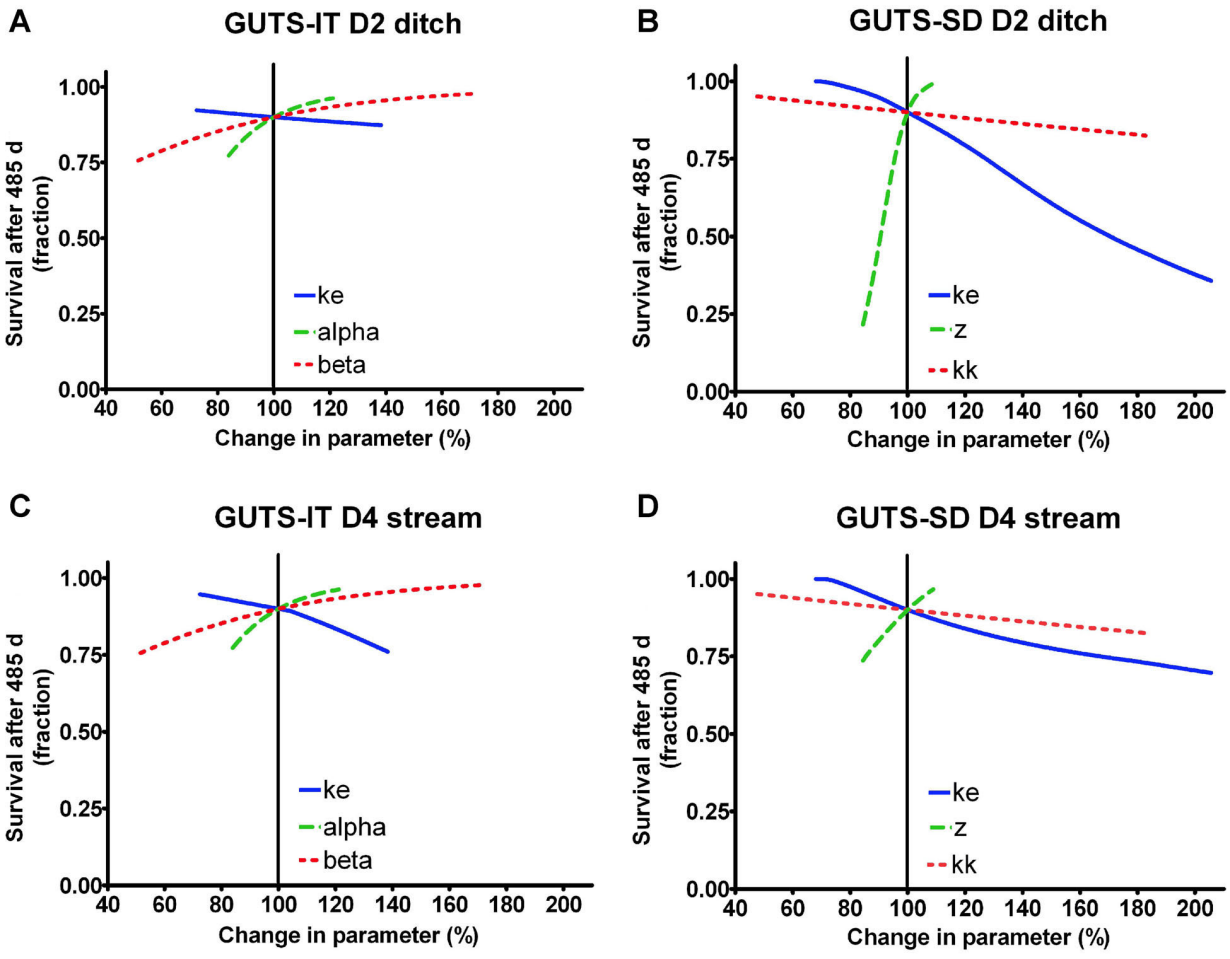
Survival after 485 d as a function of variation in single parameter values within their confidence limits. Sensitivity of the GUTS-IT model (**A**,**C**) and GUTS-SD model (**B**,**D**). The sensitivity of the model output to variation in parameter values differs for different exposure patterns (D2 ditch: upper panels, D4 stream: lower panels). See Figure 2 caption for definitions of GUTS-SD and GUTS-IT. [Color figure can be seen in the online version of this article, available at http://wileyonlinelibrary.com]

The sensitivity of the model depends on the exposure pattern and differed between GUTS-IT and GUTS-SD (Fig. 6). The sensitivity of survival after 485 d toward changes in the parameters alpha and beta of GUTS-IT were very similar in both scenarios D2 ditch and D4 stream. However, the sensitivity toward the parameters *z* and *ke* of the GUTS-SD model differed strongly between the two exposure patterns. Because each parameter was varied within its confidence limits, Figure 6 also indicates the maximum change in survival after 485 d that can be expected due to the uncertainty of a single parameter. Figure 6 clearly shows that this uncertainty also differs between parameters, model, and exposure scenario. For example, the largest change in survival after 485 d was observed for changes in parameter *z* in the GUTS-SD model and the D2 ditch exposure pattern. In D4 stream, changes in z do not lead to large changes in survival after 485 d.

## DISCUSSION

### Suitability of the model

The models GUTS-IT and GUTS-SD could be calibrated for benzovindiflupyr and used to predict fish survival following different exposure patterns. There was no clear and consistent indication regarding which of the two models, GUTS-SD or GUTS-IT, better described the acute toxicity data for all five fish species. Both models should be used, therefore, as they are the two simplified, limited cases of GUTS. Several additional calculations could be performed, such as simulations with higher exposure factors, calculations of organism recovery times and safety margins, and comparisons of exposure patterns with respect to their inherent toxic potential. Thus, compared with standard risk assessment where summary statistics of concentration–response curves are used, the models used in the present study could generate a wealth of additional information and improve our understanding of the likely relationship between fluctuating exposure patterns of benzovindiflupyr and their potential for toxic effects.

The models are calibrated with acute toxicity data from experiments of 4 d and then used to predict survival over 485 d under different exposure scenarios. To our knowledge, such an extrapolation by any model has never been tested with independent experimental data on fish survival over 485 d or similar. Within the present study, data from an ELS test with fathead minnows was used to test the predictive power of the model over 28 d. The results indicated that the model is suitable for estimating survival of fish following exposure to benzovindiflupyr.

We used a one-compartment approximation, “reduced GUTS,” of an essentially two-compartment system comprising TK and TD. The elimination rate of substances in fish depends on the size of the fish, which raises the question of whether the dominant rate constant *ke* also depends on the size of the fish. The model testing indicated that size differences between adult fathead minnow and fry do not preclude toxicity extrapolation from the adult to the fry. Nevertheless, the possible dependence of *ke* and other GUTS parameters on organism traits needs further investigation, especially for traits such as organism size that change over time and under varying field conditions.

We carried out additional simulations to determine the margins of safety for the additional three species of fish (*O. mykiss, C. variegatus,* and *L. macrochirus*) in two scenarios (lowest and highest inherent toxicity potential: D2 ditch and D4 stream, both with two applications). These simulations show that carp and fathead minnow have consistently lower margins of safety in those two fluctuating exposure profiles than the other three species (Supplemental Data). This demonstrates that carp and fathead minnow are more sensitive than the other tested fish species, not only under conditions of the LC50 toxicity test, but also under fluctuating exposure conditions.

The scaled internal concentration in our reduced GUTS model stands for both the internal concentration (TK) and the damage (TD) combined. It is important to realize that in this model, the decline of the scaled internal concentration, for example after exposure, can be slower than the actual depuration of the substance if the recovery of the organism takes longer due to lasting biochemical or physiological damage. The predictive power of this so-called reduced GUTS model is equivalent to that of the full GUTS model, which would include measured toxicokinetics 8, 19.

### Interpreting predicted survival and margins of safety

In the present case study, the simulations with the TKTD models showed that none of the tested fish would die when exposed to benzovindiflupyr over 485 d at the predicted FOCUS-SW concentrations. Of course, normal background mortality would still occur, but that was not included in the calculations in the present study because the interest was purely in the extra mortality caused by toxicant-induced mortality. Even if these exposure concentration profiles are multiplied with a factor of five, no fish are predicted to die. If the concentration profiles are multiplied with a factor 10, then approximately three of the 48 simulated combinations of fish species (carp or fathead minnow), FOCUS-SW scenario, and TKTD model (GUT-IT or GUTS-SD) would result in a maximum of 4% mortality over 485 d. The most critical scenario is D2 ditch, followed by D1 ditch. Mortality in these three cases was small; more specifically, it was 4% or less over 485 d with a safety factor of 10 applied. Assessing whether these effects could have any impact on fish populations would require an ecological perspective and would have to consider density dependence, migration, predation, and other ecological factors.

The safety margins, which allow for 10% mortality over 485 d, indicate that the concentrations in the original FOCUS-SW exposure profiles are a factor of 12 to 184 below levels for excess mortality in the analyzed combinations of fish species and application pattern. Considering that the simulations were carried out with the two most sensitive fish species (carp and fathead minnow) for benzovindiflupyr, the present study indicates that it is unlikely that fish would die due the exposure patterns as in the FOCUS-SW scenarios. The information gained by calculating the margins of safety constituted an informative additional line of evidence for environmental risk assessment. Data such as that in Figure 4 can contribute to probabilistic risk assessments.

In the present case study, we illustrate a higher-tier situation where the risk assessment was refined by testing five fish species, extensive TKTD modeling of six selected worst-case FOCUS-SW profiles, and generation of additional information such as organism recovery times, margins of safety, and comparison of the inherent toxicity potential of different exposure time series. Taken together, this information provides highly relevant further information for robustly refining the risk assessment for benzovindiflupyr.

### Model sensitivity and uncertainty

Model sensitivity depends on the parameter, model (GUTS-SD or GUTS-IT), and exposure pattern (Figure 6). The fact that model sensitivity depends on the exposure profile makes it difficult to fully understand the uncertainty of our survival predictions and generalize conclusions about model sensitivity. The model is not very sensitive to changes in some parameters, such as *alpha* and *beta* for GUTS-IT and *kk* for GUTS-SD, irrespective of which exposure pattern was used (D2 ditch or D4 stream). The model outcome is sensitive, however, to changes in *ke* (both models) and especially *z* in GUTS-SD. The sensitivity is more pronounced for D2 ditch than for D4 stream. This latter finding is related to the fact that D2 ditch contained many short pulses throughout the entire 485-d period and also a continuous raised background concentration. In contrast, D4 stream consisted of one main exposure event or exposure period only (see also Table 4). Thus, D2 ditch allows for a much stronger interplay and repeated effects of changes in *ke* and *z* on the simulated survival. This is simply because there are more peaks that can lead to exceeding the threshold *z* and more intervals between peaks that could be too short or long enough for organism recovery. The sensitivity analysis improves our understanding of the relationship between fluctuating exposure and toxic effect. However, the changes simulated in the present study that resulted from changes in one parameter at a time do not truly reflect the uncertainty in model predictions inherent in the parameters. This is the case because our one-at-a-time sensitivity analysis neglects the co-variation of parameters and, therefore, overestimates the uncertainty in model predictions. Thus, the confidence limits of the model parameters quantify the outside boundary of their uncertainty.

We did not propagate the parameter uncertainty through to the model predictions. More sophisticated modeling techniques, such as parameter estimation with Monte Carlo Markov Chains, followed by forward Monte Carlo simulations with sampling from the chain, could be used to generate prediction intervals around the simulated survival. Such simulations could capture the covariation of parameters in the model calibration and account for parameter covariation in the uncertainty analysis.

### Model testing (validation) and testing needs

The results of the model validation using the ELS data illustrate that GUTS-SD and GUTS-IT are able to predict the mortality observed in fathead minnow fry. Exposing embryos in the ELS is not modeled, and the model is calibrated to juvenile/adult fish sensitivity. Thus it might be expected that the model would predict less mortality than observed for the fry due to the general expectations that early life stages are more sensitive than juvenile and adult fish 26. As this was not the case these simulations strengthen the trust in predictions of the GUTS-SD and GUTS-IT model. Furthermore, because the model provided predictions of ELS survival, it appears that sensitivity of the two fish life stages is similar.

In addition, it must be emphasized that the models were calibrated first on acute toxicity studies and then tested by predicting the outcome of the independent data derived from the longer-term ELS study. Such a comparison (validation) is a strong test for the predictive power of a model and is not normally part of the risk assessment procedure. Overall, this validation against independent data (i.e., toxicity data not used for parameterization) provides evidence that the model is able to reliably predict effects under different (longer) exposure patterns.

In the present study, we use the GUTS models to predict survival over 485 d. It is desirable, but challenging in practice, to test the predictive capabilities of these models, or any alternative models, over such a long time span. Any alternative method of risk assessment of the FOCUS-SW exposure patterns makes an extrapolation from short-term tests to 485 d. This extrapolation step is rarely stated explicitly, although inevitable, and the underlying assumptions remain unclear and cannot be scrutinized. The assumptions of the GUTS models, however, have been clearly stated and discussed 8. Because data to test extrapolation models for fish survival over 485 d do not exist, for neither constant nor fluctuating or pulsed exposure, we cannot quantitatively evaluate the performance of any method, including GUTS.

One recent study found that different sets of calibration data result in different levels of agreement between survival data and GUTS predictions 19. That study also found that GUTS-SD and GUTS-IT performed equally well and, more importantly, that models calibrated on acute toxicity data tended to overestimate mortality under longer pulsed exposure conditions. This latter finding indicates that the method used in the present study may err on the conservative side—that is, the safe side. A forerunner model of GUTS has been calibrated and tested on independent data 14, 18. The GUTS-type TKTD model performed at least as well as the alternative models, and in a study with mixtures in time, it even predicted the effect of the sequence of exposure to two different compounds 18. The evidence available now indicates that GUTS predictions for time-variable exposure are at least as reliable and, due to the more realistic model structure, quite possibly more trustworthy than alternative models.

### Organism recovery, exposure patterns, and inherent toxic potential

The predicted exposure pattern in D2 ditch for multiple exposure events is spread throughout the entire simulation period (Fig. 5), whereas predicted exposure in D4 stream shows one major event around day 350. Multiple exposure events, for example in D2 ditch, allow the organisms to recover between pulses; thus, such a pattern has the lowest inherent toxic potential. This must not be confused with the fact that D2 ditch was the exposure pattern that is closest to the onset of mortality, as indicated by the lowest margins of safety (see Table 2). In the FOCUS-SW simulations, D2 ditch reaches comparatively high exposure concentrations. Thus, the high absolute exposure in D2 ditch overcompensates for its low inherent toxic potential.

A comparison of organism recovery times with the intervals between peaks can yield additional insight into the potential toxicity of the exposure profiles. The D4 stream scenario had the lowest number of peaks (Table 4), but none of the characteristics yielded a clear pattern identifying the exposure profile with the lowest or highest inherent toxic potential. However, comparing the mean interval between peaks (between 4 and 22 d) with the organism recovery times (between 2 and 6 d) indicates that the fish, on average, will be able to recover between peaks; that is, the exposure events can be seen as toxicologically independent. Note that D2 stream, which has the shortest mean interval between peaks (4 d) also has the shortest average peak duration (3 d), so that, on average, recovery is also plausible in the present study, even though there are 109 peaks over the period of 485 d. Comparing the D2 ditch and D4 stream also demonstrates that for benzovindiflupyr, toxicity is not simply a function of the area under the exposure curve, because the areas under the curves differ widely for different patterns that result in the same overall effect (see Table 3).

### Use in higher tier risk assessment for registration of PPPs

The use of TKTD modeling, in particular predicting survival using GUTS, supplements existing environmental risk assessment methods well because carry-over effects and delayed toxicity can be simulated. Furthermore, using the two extreme cases, GUTS-SD and GUTS-IT, increases the confidence in the risk assessments because these two generic models apply to all mechanisms of toxicity. The question regarding which margin of safety is acceptable and which percentage of mortality in simulations with certain exposure factors would be acceptable remains a risk management question. Any use of short-term toxicity data to arrive at risk assessment decisions for the 485-d FOCUS-SW exposure patterns requires assumptions and underlying models, and these are rarely stated explicitly or justified. Thus, the clear communication of underlying assumptions of GUTS 8 increases transparency and understanding of the risk assessment process. In addition, the analysis presented in the present study makes use of the raw data from the acute toxicity test; thus, it extracts more information than summary statistics such as LC50 values and facilitates extrapolations that are not possible with LC50 based predictions alone.

The toxic potency of fluctuating or pulsed exposures cannot be known a priori. Rather, the interplay of longer, low concentrations and shorter, high concentrations resulted in a non-linear relationship with toxicity, which is specific to each combination of species and test substance. As the survival prediction with GUTS can also identify which parts of the exposure profile are potentially most toxic to organisms, such analyses can also guide more targeted mitigation measures.

## CONCLUSION

Taking time-variable exposure explicitly into account via TKTD modeling improves our understanding of the relationship between fluctuating exposure and toxicity. The GUTS is currently the best tool when the endpoint is survival. The additional information and insight gained through TKTD modeling and careful analysis of the exposure patterns can strengthen the environmental risk assessment of PPPs.

## SUPPLEMENTAL DATA

Model calibration and parameter estimates for three additional fish species as well as a comparison of survival under fluctuating exposure for all five fish species.

**Tables S1 to S7**.

**Figs. S1 to S6**. (201 KB PDF).

## References

[1] Kreuger J (1998). Pesticides in stream water within an agricultural catchment in southern Sweden, 1990–1996. Sci Total Environ.

[2] Wittmer IK, Bader HP, Scheidegger R, Singer H, Lück A, Hanke I, Carlsson C, Stamm C (2010). Significance of urban and agricultural land use for biocide and pesticide dynamics in surface waters. Water Res.

[3] Brock TCM, Alix A, Brown CD, Capri E, Gottesbüren B, Heimbach F, Lythgo CM, Schulz R, Streloke M (2010). Linking Aquatic Exposure and Effects.

[4] Reinert KH, Giddings JA, Judd L (2002). Effects analysis of time-varying or repeated exposures in aquatic ecological risk assessment of agrochemicals. Environ Toxicol Chem.

[5] Ashauer R, Boxall ABA, Brown CD (2006). Predicting effects on aquatic organisms from fluctuating or pulsed exposure to pesticides. Environ Toxicol Chem.

[6] Ashauer R, Wittmer I, Stamm C, Escher BI (2011). Environmental risk assessment of fluctuating diazinon concentrations in an urban and agricultural catchment using toxicokinetic-toxicodynamic modeling. Environ Sci Technol.

[8] Jager T, Albert C, Preuss TG, Ashauer R (2011). General unified threshold model of survival: A toxicokinetic-toxicodynamic framework for ecotoxicology. Environ Sci Technol.

[9] Ashauer R, Escher BI (2010). Advantages of toxicokinetic and toxicodynamic modelling in aquatic ecotoxicology and risk assessment. J Environ Monitor.

[10] Jager T (2011). Some good reasons to ban ECx and related concepts in ecotoxicology. Environ Sci Technol.

[11] Jager T, Heugens EHW, Kooijman SALM (2006). Making sense of ecotoxicological test results: Towards application of process-based models. Ecotoxicology.

[12] Ashauer R, Agatz A, Albert C, Ducrot V, Galic N, Hendriks J, Jager T, Kretschmann A, O'Connor I, Rubach MN, Nyman A-M, Schmitt W, Stadnicka J, Van den Brink PJ, Preuss TG (2011). Toxicokinetic-toxicodynamic modeling of quantal and graded sublethal endpoints: A brief discussion of concepts. Environ Toxicol Chem.

[13] Ashauer R, Hintermeister A, Caravatti I, Kretschmann A, Escher BI (2010). Toxicokinetic-toxicodynamic modeling explains carry-over toxicity from exposure to diazinon by slow organism recovery. Environ Sci Technol.

[14] Ashauer R, Boxall ABA, Brown CD (2007). New ecotoxicological model to simulate survival of aquatic invertebrates after exposure to fluctuating and sequential pulses of pesticides. Environ Sci Technol.

[15] Stadnicka J, Schirmer K, Ashauer R (2012). Predicting concentrations of organic chemicals in fish by using toxicokinetic models. Environ Sci Technol.

[16] Hommen U, Ashauer R, Van den Brink P, Caquet T, Ducrot V, Lagadic L, Ratte HT, Brock TCM, Alix A, Brown CD, Capri E, Gottesbüren B, Heimbach F, Lythgo CM, Schulz R, Streloke M (2010). Extrapolation methods in aquatic effects assessment of time-variable exposures to pesticides. Linking Aquatic Exposure & Effects in the Risk Assessment of Pesticides.

[17] Altenburger R, Greco WR (2009). Extrapolation concepts for dealing with multiple contamination in environmental risk assessment. Integr Environ Assess Manag.

[18] Ashauer R, Boxall ABA, Brown CD (2007). Modeling combined effects of pulsed exposure to carbaryl and chlorpyrifos on *Gammarus pulex*. Environ Sci Technol.

[19] Nyman A-M, Schirmer K, Ashauer R (2012). Toxicokinetic-toxicodynamic modelling of survival of *Gammarus pulex* in multiple pulse exposures to propiconazole: Model assumptions, calibration data requirements and predictive power. Ecotoxicology.

[20] Ashauer R (2010). Toxicokinetic-toxicodynamic modelling in an individual based context—Consequences of parameter variability. Ecol Model.

[21] Zhao Y, Newman MC (2007). The theory underlying dose–response models influences predictions for intermittent exposures. Environ Toxicol Chem.

[22] Kooijman SALM, Bedaux JJM (1996). Some statistical properties of estimates of no-effect concentrations. Water Res.

[24] Ashauer R, Boxall ABA, Brown CD (2007). Simulating toxicity of carbaryl to *Gammarus pulex* after sequential pulsed exposure. Environ Sci Technol.

[25] Ashauer R, Brown CD (2007).

[26] McKim JM (1977). Evaluation of tests with early life stages of fish for predicting long-term toxicity. J Fish Res Board Can.

