# Supplementary material for: A METHOD TO PREDICT AND UNDERSTAND FISH SURVIVAL UNDER DYNAMIC CHEMICAL STRESS USING STANDARD ECOTOXICITY DATA
Source: Environ Toxicol Chem. 2013 Jan 30;32(4):954–65. doi: 10.1002/etc.2144 (PMC3615168; doi:10.1002/etc.2144)
Supplement: Supplementary file 1 [file etc0032-0954-SD1.pdf]

Supporting information

to

A method to predict and understand fish survival under dynamic chemical stress using standard ecotoxicity data

Roman Ashauer [†, ‡], Pernille Thorbek [§], Jacqui S Warinton [§], James R Wheeler [§] and Steve Maund[||]

†) Gumpisbüelstrasse 60, 8600 Dübendorf, Switzerland

‡) Eawag, Swiss Federal Institute of Aquatic Science and Technology, Überlandstrasse 133, 8600 Dübendorf, Switzerland

§) Syngenta Ltd, Environmental Safety, Jealott's Hill, Bracknell, Berkshire, RG42 6EY, United Kingdom

||) Syngenta Crop Protection AG, Schwarzwaldallee 215, 4058 Basel, Switzerland

Corresponding author: Roman Ashauer

Email: roman@ecotoxmodels.org

URL: www.ecotoxmodels.org

Table of contents

Model calibration 2
Calibration: rainbow trout (Oncorhynchus mykiss) 2
Calibration discussion: rainbow trout 4
Calibration: sheepshead minnow (Cyprinodon variegatus) 5
Calibration discussion: sheepshead minnow 6
Calibration: bluegill sunfish (Lepomis macrochirus) 7
Calibration discussion: bluegill sunfish 8
Comparing sensitivity of fish species 9

Model calibration

Calibration: rainbow trout (Oncorhynchus mykiss)

GUTS-SD

The best fit of the GUTS-SD model to the acute toxicity data was obtained with -∑ln *likelihood* = 9.64208 and the parameter values in Table S1. Background mortality: *h_controls* = 0.0035 [1/d].

Table S1: Best fit parameter values for the GUTS-SD model (rainbow trout)

Parameter	Units	Best fit value	95% confidence limits
ke	1 / d	99.91	[10; n.d.] [a)]
kk	L / (µg × d)	5.96	[1.78; 13.90]
z	µg / L	9.75	[9.69; 9.79]

a) The parameter *ke* converged to its upper constraint (limit 100). The upper confidence limit could not be determined (n.d. = not determined). There is only one treatment (9.8 µg / L) with partial mortality data, whereas all fish died immediately in the highest concentration. This combination is not ideal for parameter estimation and drives the parameter *ke* towards its upper constraint.

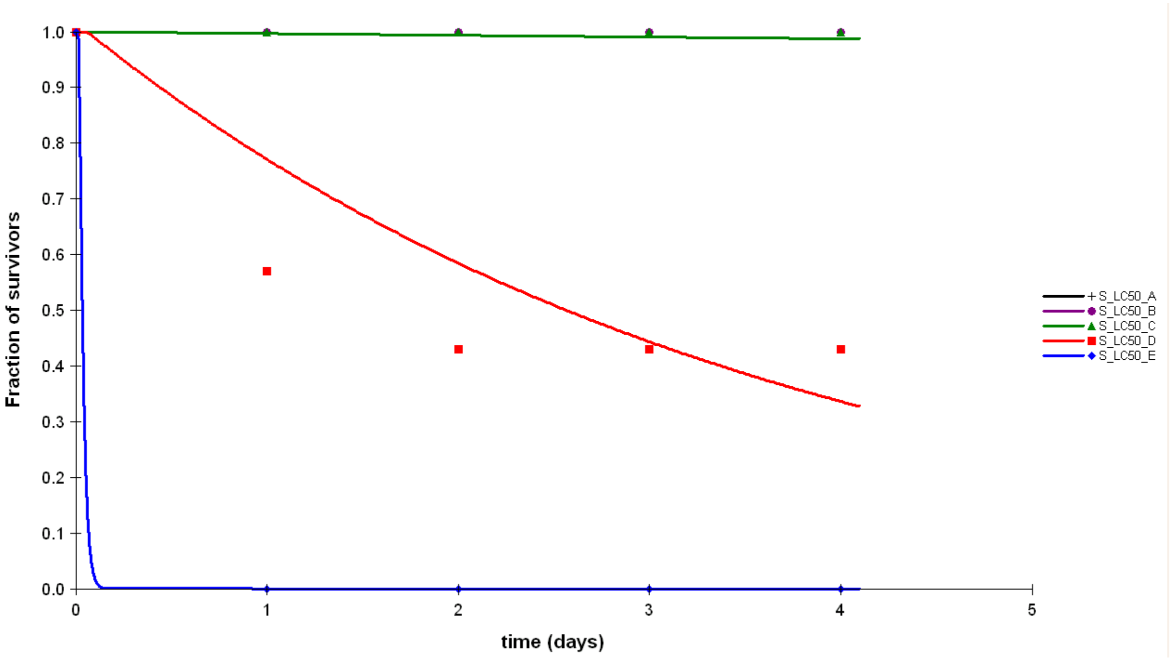

Figure S1: Fit of GUTS-SD to the acute toxicity data for rainbow trout.

Treatments D (9.8 µg/L, red squares) and E (18 µg/L, blue diamonds) show mortality.

GUTS-IT

The best fit of the GUTS-IT model to the acute toxicity data was obtained with -∑ln *likelihood* = 7.26112 and the parameter values in Table S2. Background mortality: *h_controls* = 0.0035 [1/d].

Table S2: Best fit parameter values for the GUTS-IT model (rainbow trout)

Parameter	Units	Best fit value	95% confidence limits
ke	1 / d	3.30	[2.12; 6.08]
α	µg / L	14.72	[2.90; 56.33]
β	-	9.61	[8.72; 10.61]

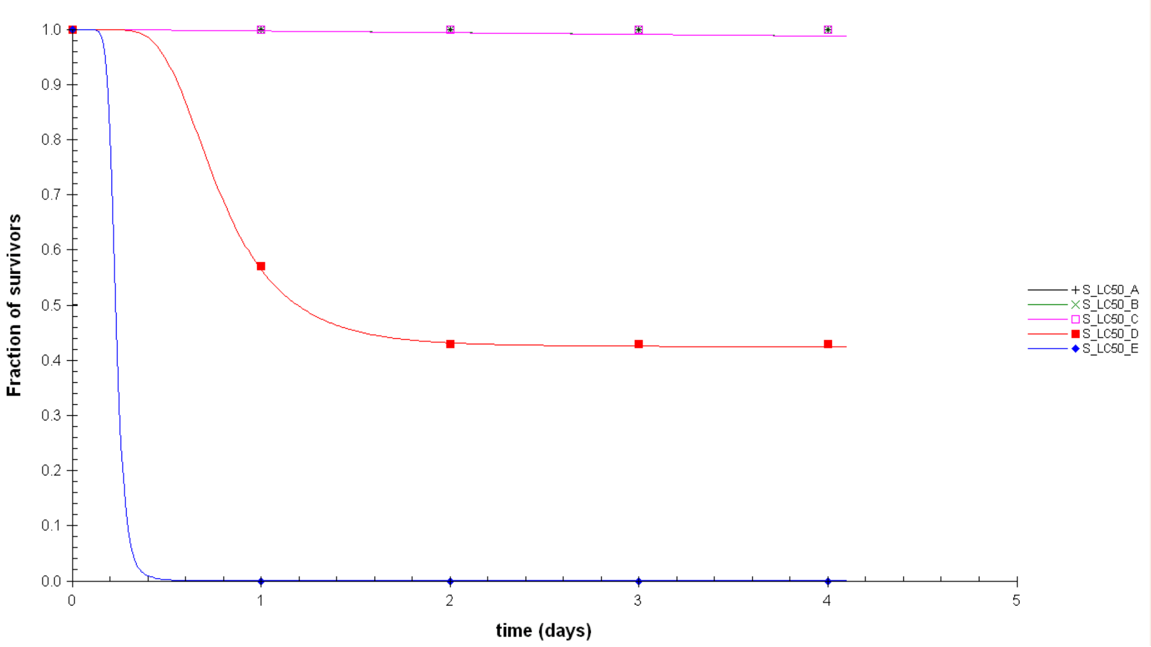

Figure S2: Fit of GUTS-IT to the acute toxicity data for rainbow trout.

Treatments D (9.8 µg/L, red squares) and E (18 µg/L, blue diamonds) show mortality.

Calibration discussion: rainbow trout

The GUTS-IT model fitted better to the rainbow trout data than the GUTS-SD model and the GUTS-IT parameters could be identified without the need for constrains. Both models result in high values for *ke*, indicating that rainbow trout have fast elimination and organism recovery capabilities with respect to this compound.

Calibration: sheepshead minnow (Cyprinodon variegatus)

GUTS-SD

The best fit of the GUTS-SD model to the acute toxicity data was obtained with -∑ln *likelihood* = 8.61931 and the parameter values in Table S3. Background mortality: *h_controls* = 0.0035 [1/d].

Table S3: Best fit parameter values for the GUTS-SD model (sheepshead minnow)

Parameter	Units	Best fit value	95% confidence limits
ke	1 / d	0.56	[0.51; 0.62]
kk	L / (µg × d)	1.12	[0.47; 2.31]
z	µg / L	26.35	[25.30; 27.26]

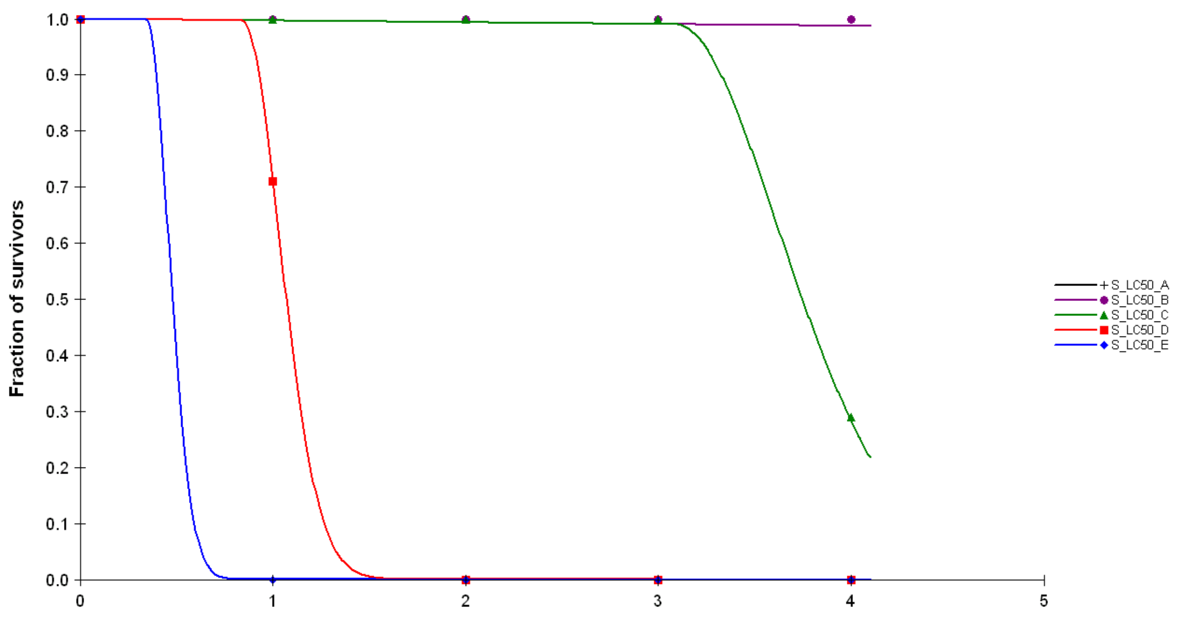

Figure S3: Fit of GUTS-SD to the acute toxicity data for sheepshead minnow.

Treatments C (32 µg/L, green triangles), D (70 µg/L, red squares) and E (150 µg/L, blue diamonds) show mortality.

GUTS-IT

The best fit of the GUTS-IT model to the acute toxicity data was obtained with -∑ln *likelihood* = 11.7271 and the parameter values in Table S4. Background mortality: *h_controls* = 0.0035 [1/d].

Table S4: Best fit parameter values for the GUTS-IT model (sheepshead minnow)

Parameter	Units	Best fit value	95% confidence limits
ke	1 / d	0.28	[0.23; 0.32]
α	µg / L	19.75	[18.05; 22.60]
β	-	9.55	[6.05; 25.78]

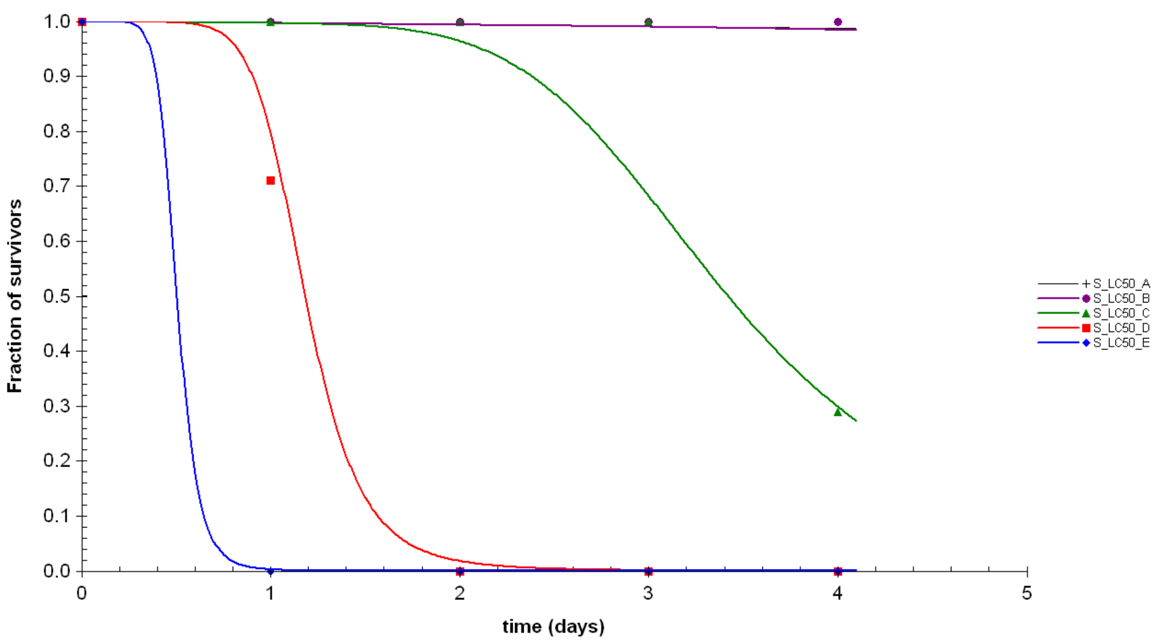

Figure S4: Fit of GUTS-IT to the acute toxicity data for sheepshead minnow.

Treatments C (32 µg/L, green triangles), D (70 µg/L, red squares) and E (150 µg/L, blue diamonds) show mortality.

Calibration discussion: sheepshead minnow

GUTS-SD fits the sheepshead minnow data better than GUTS-IT, although both models fit well. Parameter values are plausible.

Calibration: bluegill sunfish (Lepomis macrochirus)

GUTS-SD

The best fit of the GUTS-SD model to the acute toxicity data was obtained with -∑ln *likelihood* = 10.9761 and the parameter values in Table S5. Background mortality: *h_controls* = 0.0035 [1/d].

Table S5: Best fit parameter values for the GUTS-SD model (bluegill sunfish)

Parameter	Units	Best fit value	95% confidence limits
ke	1 / d	3.21	[1.86; 21.48]
kk	L / (µg × d)	0.106	[0.052; 0.204]
z	µg / L	23.78	[21.76; 24.73]

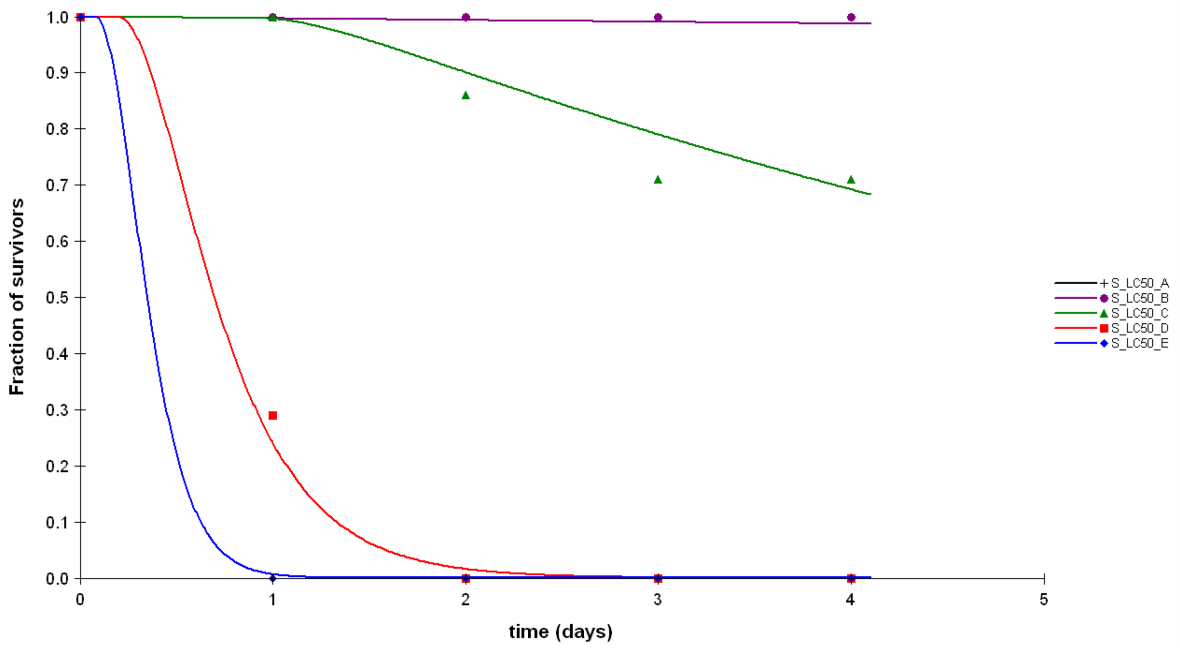

Figure S5: Fit of GUTS-SD to the acute toxicity data for bluegill sunfish.

Treatments C (25 µg/L, green triangles), D (50 µg/L, red squares) and E (100 µg/L, blue diamonds) show mortality.

GUTS-IT

The best fit of the GUTS-IT model to the acute toxicity data was obtained with -∑ln *likelihood* = 13.194 and the parameter values in Table S6. Background mortality: *h_controls* = 0.0035 [1/d].

Table S6: Best fit parameter values for the GUTS-IT model (bluegill sunfish)

Parameter	Units	Best fit value	95% confidence limits
ke	1 / d	0.85	[0.67; 1.11]
α	µg / L	25.57	[23.02; 28.70]
β	-	10.04	[4.43; 19.65]

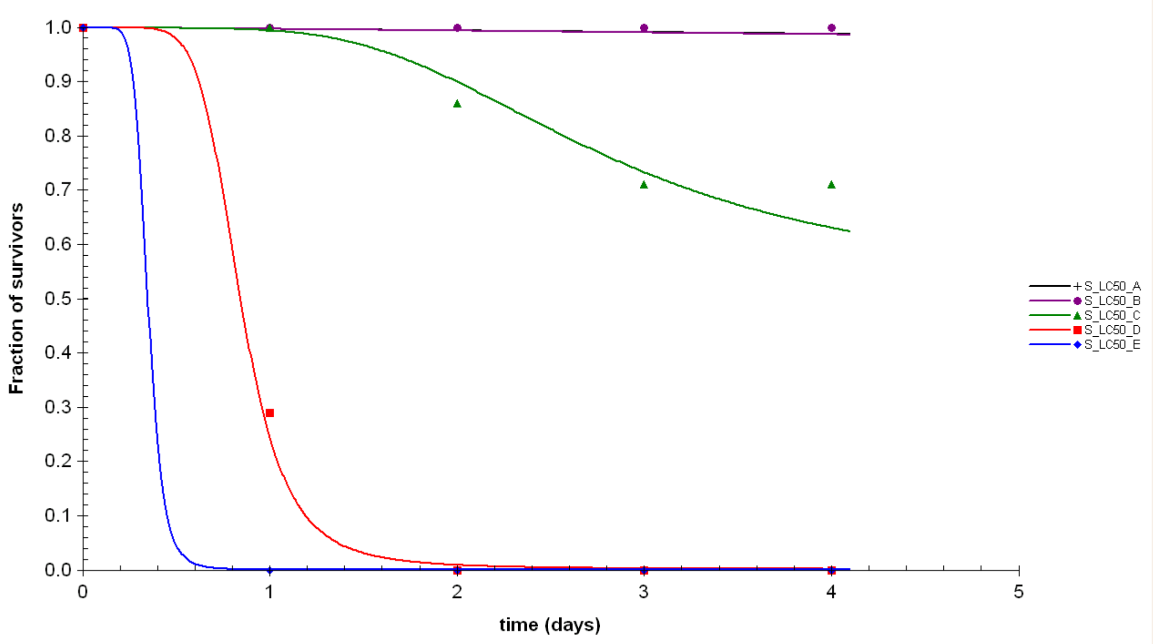

Figure S6: Fit of GUTS-IT to the acute toxicity data for bluegill sunfish.

Treatments C (25 µg/L, green triangles), D (50 µg/L, red squares) and E (100 µg/L, blue diamonds) show mortality.

Calibration discussion: bluegill sunfish

Both, GUTS-IT and GUTS-SD fit the data well. Parameter values are plausible. Note that for GUTS-SD the parameter *ke* has large confidence limits.

Comparing sensitivity of fish species

We calculate the margins of safety for all five fish species in scenarios D2 ditch (2 applications) and D4 stream (2 applications). The shape of the concentration time series in D2 ditch has the least inherent toxicity and D4 stream the most inherent toxicity for both, carp and fathead minnow. The safety margins, i.e. the exposure multiplication factors leading to 90% survival after 485 days, are shown in Table S7. Lower values indicate a sensitive species, higher values a less sensitive species.

Table S7: Comparison of safety margins for five fish species.

**Scenario**	**Model (GUTS)**	**D2 ditch 2 applications**	**D4 stream 2 applications**
Carp	SD	16.3	80.0
	IT	13.2	82.7
Fathead minnow	SD	15.3	74.7
	IT	11.9	74.9
Rainbow trout	SD	21.1	88.5
	IT	32.5	140.8
Sheepshead minnow	SD	116.1	681.7
	IT	75.9	546.7
Bluegill sunfish	SD	76.2	340.9
	IT	82.4	412.4
